# Short-term preoperative drainage is associated with improved postoperative outcomes compared to that of long-term biliary drainage in pancreatic surgery

**DOI:** 10.1007/s00423-021-02402-7

**Published:** 2021-12-15

**Authors:** F. Oehme, S. Hempel, M. Pecqueux, B. Müssle, H. M. Hau, C. Teske, F. von Bechtolsheim, L. Seifert, M. Distler, T. Welsch, J. Weitz, Cristoph Kahlert

**Affiliations:** Department for Visceral-, Thoracic and Vascular Surgery, University Hospital Carl Gustav Carus, Technische Universität Dresden, Fetscherstrasse 74, 01307 Dresden, Germany

**Keywords:** Preoperative biliary drainage, Pancreatic cancer, Pancreatic surgery, Complication

## Abstract

**Purpose:**

The treatment of choice for patients presenting with obstructive cholestasis due to periampullary carcinoma is oncologic resection without preoperative biliary drainage (PBD). However, resection without PBD becomes virtually impossible in patients with obstructive cholangitis or severely impaired liver cell function. The appropriate duration of drainage by PBD has not yet been defined for these patients.

**Methods:**

A retrospective analysis was conducted on 170 patients scheduled for pancreatic resection following biliary drainage between January 2012 and June 2018 at the University Hospital Dresden in Germany. All patients were deemed eligible for inclusion, regardless of the underlying disease entity. The primary endpoint analysis was defined as the overall morbidity (according to the Clavien-Dindo classification). Secondary endpoints were the in-hospital mortality and malignancy adjusted overall and recurrence-free survival rates.

**Results:**

A total of 170 patients were included, of which 45 (26.5%) and 125 (73.5%) were assigned to the short-term (< 4 weeks) and long-term (≥ 4 weeks) preoperative drainage groups, respectively.

Surgical complications (Clavien-Dindo classification > 2) occurred in 80 (47.1%) patients, with significantly fewer complications observed in the short-term drainage group (31.1% vs. 52%; *p* = 0.02). We found that long-term preoperative drainage (unadjusted OR, 3.386; 95% CI, 1.507–7.606; *p* < 0.01) and periampullary carcinoma (unadjusted OR, 5.519; 95% CI, 1.722–17.685; *p*-value < 0.01) were independent risk factors for postoperative morbidity, based on the results of a multivariate regression model. The adjusted overall and recurrence-free survival did not differ between the groups (*p* = 0.12).

**Conclusion:**

PBD in patients scheduled for pancreatic surgery is associated with substantial perioperative morbidity. Our results indicate that patients who have undergone PBD should be operated on within 4 weeks after drainage.

## Background

Preoperative biliary drainage (PBD) in patients presenting with painless jaundice due to periampullary carcinoma has been an issue of debate for many years. According to van der Gaag et al. [1], surgical resection without preoperative treatment of cholestasis by drainage should be the treatment of choice for periampullary carcinoma with concomitant jaundice [1–4]. However, surgery without PBD in patients with cholangitis, severely impaired liver cell function, or those undergoing neoadjuvant treatment is virtually impossible [5]. These patients usually require PBD to relieve cholestasis and its detrimental effects [6]. Some of the well-described effects include hepatic inflammation with liver cell damage, renal dysfunction, bacteriobilia, and imbalanced T-cell homeostasis [6–8]. Experimental data suggest that hepatic function recovers after a minimum of 4–6 weeks following PBD [9]. However, there is a paucity of clinical data on this issue.

Interestingly, the existing data for the subcohort of patients with PBD are weak and somewhat inconsistent with respect to the accurate preoperative drainage time. Son et al. [10] found that a biliary drainage duration of less than 2 weeks might be sufficient and correlated with a favorable outcome following pancreatic resection. However, Sandini et al. [11] suggested that a PBD time of more than 4 weeks might be superior in terms of postoperative morbidity rates.

Thus, it remains inconclusive whether patients develop more postoperative complications following a short or long biliary drainage duration prior to pancreaticoduodenectomy. In addition, there is still a lack of sufficient data to determine the clinical benefit of prophylactic stenting in patients with tumor-related cholestasis who are scheduled for neoadjuvant chemotherapy.

Therefore, the primary aim of this study was to evaluate the optimal period for PBD prior to pancreatic resection. We aimed to provide data on perioperative morbidity and mortality rates, along with overall and recurrence-free survival rates.

## Methods

This article was written in accordance with the STROBE statement [12]. The experimental protocol was approved by the local ethics committee of the Technische Universität (TU) Dresden (decision number EK575122019). All the methods were conducted in accordance with the relevant guidelines. This study was a retrospective case series of all patients who underwent surgery between January 2012 and June 2018 at the Department of Visceral-, Thoracic- and Vascular Surgery, University Hospital Carl Gustav Carus, TU Dresden, Germany. Patients were eligible for inclusion if they had undergone pancreatic resection for pancreatic and periampullary carcinoma, cystic pancreatic lesions, or chronic pancreatitis following biliary drainage.

No exclusion criteria were defined regarding the time between drainage and surgery, the entity of the underlying disease, or the type of operation performed.

Basic patient characteristics included median age, sex, body mass index (BMI), and physical status according to the American Society of Anesthesiologists (ASA) [13]. The patients who were included presented with the following diagnoses: chronic pancreatitis, pancreatic ductal adenocarcinoma (PDAC), neuroendocrine tumor (NET), or distal bile duct adenocarcinoma. The types of resections performed included partial pylorus-preserving pancreaticoduodenectomy (PPPD), classic pancreaticoduodenectomy (cPD), duodenum-preserving pancreaticoduodectomy (PD), and total pancreatectomy (TP). All medical records were extracted from electronic patient files.

### Indications and patient management of preoperative biliary drainage

PBD is usually avoided unless there is no alternative due to patient-related or therapy-associated factors. Indications for PBD include excessive jaundice (> 250 µmol/L) with severe symptoms (e.g., pruritus, acute renal failure and c-reactive protein > 150 mg/l), neoadjuvant treatment, acute obstructive cholangitis, or impaired liver cell function [5]. The standard intervention plan in our department for patients with these indications is drainage via endoscopic retrograde cholangiopancreatography and plastic stent placement whenever possible (usually 8–12 French stents).

The preoperative period between stent placement and surgery was classified as one of two categories: < 4 weeks (PBD I) and > 4 weeks (PBD II). The groups were defined according to the most recent literature [11].

### Primary endpoint

The primary endpoint for this study was the overall morbidities defined according to the Clavien–Dindo classification (CDC) [14] that are classified as major complications (CDC > 2), including delayed gastric emptying (DGE) [15], postoperative pancreatic fistula (POPF) [16] and post-pancreatectomy hemorrhage (PPH) [17], which were defined and graded according to the consensus definitions of the International Study Group of Pancreatic Surgery.

Morbidity analysis was performed separately for the different PBD periods.

### Secondary endpoint

Data on the length of hospital stay (LOS) and duration of intensive care unit stay were extracted from the patient files. The rehospitalization rate was defined as any hospital readmission within 30 days of discharge after the index operation due to PD-related complications. Overall survival (OS) and recurrence-free survival (RFS) rates were adjusted for in-hospital mortality and underlying malignancy. The adjusted OS for patients with an underlying malignancy was calculated as the date of death or the time of last contact (censored) and RFS, by the date of diagnosis of recurrence.

### Bile duct culture

Intraoperative bile duct swabs were analyzed with respect to the frequency of positive bacterial detection. In addition, the resistance situation of the bacterial cultures found in the bile duct swabs was analyzed according to the publication by Magiorakos et al. [18].

The classification of the results was made according to the observed results of the bile duct cultures: negative bile duct cultures, multidrug-sensitive (MDS), multidrug-resistant (MDR), and extensivedrug-resistant (XDR).

### Statistical analysis

Data were analyzed using SPSS version 21 software (IBM Corp., Armonk, NY, USA). The normality of continuous data was assessed using the Kolmogorov–Smirnov test and by inspecting the frequency distributions. Variance homogeneity was tested using Levene’s test.

Competitive analysis was performed to compare baseline characteristics between the groups of patients categorized according to PBD duration using the chi-square test or Fisher’s exact test for categorical variables. Unifactorial analysis of variance, Student’s *t*-test, or the Mann–Whitney *U* test was performed on continuous variables where appropriate, and the results were represented as median and interquartile range (IQR).

Logistic regression analysis was performed to determine the relationship between the different groups regarding PBD duration and CDC > 2 complications. Other factors included in the multivariate regression analysis were comorbidities, preoperative cholestasis, and preoperative chemotherapy. All clinically relevant variables and those with a p-value of < 0.3 in univariate regression analysis were included in a multivariate stepwise regression model.

The Kaplan–Meier method was used to calculate OS and RFS curves, and the log-rank test was used to identify the differences between the curves.

A *p*-value of < 0.05 was considered the threshold of statistical significance for all analyses. During the analyses, missing data were treated as *missing completely at random*. Thus, a complete case analysis was performed, and some patients were excluded from the analysis.

## Results

### Basic patient characteristics

Between January 2012 and June 2018, a total of 702 patients scheduled for pancreatic resection due to an underlying malignancy, borderline tumor (intraductal papillary mucinous neoplasms), or chronic pancreatitis were identified using data from a prospectively created database.

Patients without PBD were excluded from further analysis (*n* = 532). Our study cohort included a total of 170 (24.2%) patients with PBD with a median age of 68.2 years. Of these 170 patients, 149 (87.6%) underwent surgery for an underlying malignancy. Pancreatic ductal adenocarcinoma (PDAC), neuroendocrine tumor (NET), and distal bile duct malignancies were histologically confirmed in 71 (41.8%), 4 (2.4%), and 74 (43.5%) cases, respectively. The operations performed were PPPD, cPD, DPPHR, and TP in 112 (65.9%), 29 (17.1%), 8 (4.7%), and 18 (10.6%) cases, respectively.

Further characteristics of the cohort in terms of comorbidities and the operations performed are shown in Table 1.

**Table 1:** Basic patient characteristics

	Overall	Short (PBD I)	Long (PBD II)	*p*-value
Patients [*n* (%)]	170	45 (26.5)	125 (73.5)	
Sex [*n* (%)]				
M	106 (62.4)	26 (57.8)	80 (64)	0.46
W	64 (37.6)	19 (42.2)	45 (36)	
Median age [years] (IQR)	68.2 (61.7–76.8)	71.2 (63.7–75.8)	68 (60.6–77.2)	0.43
Median BMI [kg/m^2^] (IQR)	24.4 (22.2–27.2)	24.4 (22.1–27.1)	24.5 (22.4–27.3)	0.68
Preoperative bilirubin [mmol/l] (IQR)	17.2 (9.3–43.3)	35.3 (17.8–67.3)	13.9 (7.5–28.6)	** < 0.01**
Preoperative cholestasis [*n* (%)]	77 (45.3)	32 (71.1)	45 (36)	** < 0.001**
Preoperative CA 19–9 [U/ml] (IQR)	72.8 (13.2–270.8)	98.1 (39.9–211.3)	51.8 (12.7–277.8)	0.15
preoperative CEA [U/ml] (IQR)	2.2 (1.4–3.4)	2.1 (1.2–3.4)	2.3 (1.4–3.4)	0.64
*ASA score [n (%)]*				
1	8 (4.7)	1 (2.2)	7 (5.6)	0.72
2	66 (39.1)	19 (42.2)	47 (37.9)	
3	94 (55.6)	25 (55.6)	69 (55.6)	
4	1 (0.6)	0	1 (0.8)	
Diabetes [*n* (%)]	66 (38.8)	20 (44.4)	46 (36.8)	0.37
Insulin-dependent diabetes (IDDM) [*n* (%)]	38 (22.4)	12 (26.7)	26 (20.8)	0.42
Neoadjuvant chemotherapy [*n* (%)]	13 (7.6)	0	13 (10.4)	**0.02**
*Histopathological analysis [n (%)]*				
Pancreatitis	21 (12.4)	1 (2.2)	20 (16)	0.07
Pancreatic ductal adenocarcinoma (PDAC)	71 (41.8)	24 (53.3)	47 (37.6)	
Pancreatic neuroendocrine tumor (NET)	4 (2.4)	1 (2.2)	3 (2.4)	
Distal bile duct malignancy	74 (43.5)	19 (42.2)	55 (44)	
*Operations performed [n (%)]*				
Partial pylorus-preserving PD (PPPD)	112 (65.9)	34 (75.6)	78 (62.4)	0.12
Classic PD (cPD)	29 (17.1)	5 (11.1)	24 (19.2)	
Duodenum-preserving PD	8 (4.7)	0	8 (6.4)	
Total pancreatectomy (TP)	18 (10.6)	6 (13.3)	12 (9.6)	
Others	3 (1.7)	0	3 (2.4)	
Intraoperative blood loss [ml] (IQR)	525 (400–900)	600 (450–1000)	500 (400–825	0.34
FRS–fistula risk score (IQR)	2 (1–3)	2 (1–3)	2 (1–3)	0.15

Bolded text signifies significant findings

### Cohort exploration–preoperative morbidity

Of the 170 patients included in the study, 45 were assigned to the short-term group (< 4 weeks; PBD I) and 125 into the long-term group (≥ 4 weeks; PBD II). Both groups were comparable in terms of sex distribution, median age, BMI, preoperative tumor marker, ASA score, and insulin/non-insulin-dependent diabetes (Table 1).

The incidence of preoperative cholestasis differed significantly between groups. The percentage of patients in the short- and long-term groups who presented with hyperbilirubinemia at the time of surgery was 71.1% and 36% (p < 0.001), respectively.

The median time between preoperative stent placement and surgery was 40.5 days (IQR 27–90.3), with the majority of stents being plastic stents (95.3%). Stent replacement was performed in 43 (25.3%) cases and at least twice in 25 (14.7%) cases. Patients in the long-term group underwent significantly (p < 0.001) more stent replacements. In the short-term group, only six patients (13.3%) required a stent replacement. Additional stent-related information is presented in Table 2.

**Table 2:** Biliary drainage–indication/duration

	Overall
Mean duration of preoperative stent placement (days) (IQR)	40.5 (27–90.3)
Preoperative cholestasis [*n* (%)]	77 (45.3)
*Duration of preoperative stent placement [n (%)]*	
< 4 weeks	45 (26.5)
> 4 weeks	125 (73.5)
*Preoperative stent replacement [n (%)]*	
0	99 (58.2)
1	43 (25.3)
> 1	25 (14.7)
*Type of stent [n (%)]*	
Plastic	162 (95.2)
Metal	4 (2.4)
Combination	4 (2.4)

Patients who underwent surgery for pancreatitis experienced a significantly (*p* < 0.001) longer time interval between stent placement and surgery (median = 105 days; IQR: 51–202) compared to patients with PDAC (median = 40 days; IQR: 25–74), NET (median = 52 days; IQR: 20.5–136), and distal bile duct carcinoma (median = 37.5 days; IQR: 27–65.5).

### Bile duct culture

The results of the bile duct culture were available in 127 (74.7%) of the cases, of which 103 (81.1%) showed a positive smear test. The short-term stenting group had significantly (*p*-value < 0.001) fewer positive bile duct cultures (46.7%) compared to the long-term stenting group (91.8%).

The same observation could be seen with regard to the resistancy pattern: Significantly more MDR and XDR bacteria were found in the PBDII group (Table 3).

**Table 3:** Intraoperative bile duct cultures-resistancy pattern

	Overall	Short (PBD I)	Long (PBD II)	*p*-value
Bile duct cultures positive [*n* (%)]	103 (81.1)	14 (46.7)	89 (91.8)	** < 0.001**
*Drug resistancy [n (%)]*				
Negative	24 (18.9)	16 (53.3)	8 (8.2)	** < 0.001**
MDS	48 (37.8)	8 (26.7)	36 (37.1)	
MDR	49 (38.6)	5 (16.7)	47 (48.5)	
XDR	6 (4.7)	1 (3.3)	6 (6.2)	

Negative: negative bile duct cultures; *MDS* multidrug-sensitive, *MDR* multidrug-resistant, XDR extensive drug-resistant

Bolded text signifies significant findings

### Primary endpoint

Overall, major complications occurred in 80 patients (47.1%), and the complication rate differed significantly (*p* = 0.02) between the PBD groups. Patients assigned to the short-term PBD group experienced significantly fewer complications (31.1%) than those in the long-term PBD group (52%; *p* = 0.02). The results are presented in Table 4.

**Table 4:** Outcome analysis

	Overall	Short (PBD I)	Long (PBD II)	*p*-value
Patients [*n* (%)]	170	45 (26.5)	125 (73.5)	
Length of hospital stay (LOS) (IQR)	21 (14–29.3)	19 (14–24)	22 (15–30)	**0.02**
Length of intensive care unit stay (ICU stay) (IQR)	3 (2–6.5)	3 (2–4)	4 (2–5.5)	0.07
30-day readmission rate [*n* (%)]	24 (13.5)	4 (8.9)	20 (16)	0.24
Adjuvant CTX received (if indicated) [n (%)]	59 (39.6)	23 (51.2)	36 (35.3)	0.08
*Postoperative complication [n (%)]*				
CDC > 2 [*n* (%)]	80 (47.1)	14 (31.1)	66 (52)	**0.02**
In-hospital mortality [*n* (%)]	15 (8.8)	1 (2.2)	14 (11.2)	0.07
30-day mortality [*n* (%)]	13 (8.7)	2 (4.5)	11 (8.8)	0.24
90-day mortality [*n* (%)]	20 (11.8)	4 (8.9)	16 (12.8)	0.32
Surgical site infection (SSI) [*n* (%)]	47 (27.7)	9 (20)	38 (30.4)	0.18
Fascial dehiscence [*n* (%)]	12 (7.1)	1 (2.2)	11 (8.8)	0.18
Delayed gastric emptying (DGE) [*n* (%)]	33 (19.4)	10 (22.2)	23 (18.4)	0.58
Pancreatic fistula (POPF) [*n* (%)]	27 (15.9)	5 (11.1)	22 (17.6)	0.31
Postpancreatectomy hemorrhage (PPH) [*n* (%)]	19 (11.2)	4 (8.9)	15 (12)	0.57

Bolded text signifies significant findings

According to results obtained using univariate regression analysis, patients with long-term drainage had an increased risk (odds ratio (OR) 2.399; 95% confidence interval (CI), 1.165–4.939; *p* = 0.02) for major postoperative complications (CDC > 2). The *p*-value threshold of 0.3 in univariate regression analysis was reached in patients with an ASA score II (OR 1.479; 95% CI, 0.789–2.750; *p* = 0.2), ASA-score III (OR 1.803; 95% CI, 0.973–3.339; *p* = 0.06), ductal adeno-carcinoma (OR 2.158; 95% CI, 0.712–6.543; *p* = 0.2), neuroendocrine tumor (OR 3.2; 95% CI, 0.354–28.945; *p* = 0.3), periampullary carcinoma (OR 4.8; 95% CI, 1.589–14.497; *p* = 0.01), and preoperative anemia (OR 1.375; 95% CI, 0.748–2.528; *p* = 0.3) (Table 5).

**Table 5:** Univariate and multivariate regression analysis

	Univariate analysis			Multivariate analysis		
	Relative OR	95% CI	*p*-value	Relative OR	95% CI	*p*-value
*Duration of stent placement*						
Long (PBD II)	2.399	1.165–4.939	0.02	3.386	1.507–7.606	** < 0.01**
*Preoperative morbidity*						
ASA 2	1.473	0.789– 2.750	0.22	3.1	0.568–16.901	0.19
ASA 3	1.803	0.973–3.339	0.06	4.532	0.851–24.144	0.8
Preoperative cholestasis	1.236	0.674–2.265	0.5	1.278	0.634–2.577	0.49
Preoperative anemia	1.375	0.748–2.528	0.31	1.605	0.804–3.203	0.18
Neoadjuvant chemotherapy	0.701	0.220–2.237	0.55	0.631	0.182–2.189	0.47
*Histology*						
Benign	Reference					
Ductal adeno-carcinoma	2.158	0.712–6.543	0.17	3.003	0.905–9.964	0.07
Neuroendocrine tumor	3.2	0.354–28.945	0.3	4.515	0.477–42.763	0.19
Periampullary carcinoma	4.8	1.589–14.497	0.01	5.519	1.722–17.685	** < 0.01**
Preoperative billirubin value	0.998	0.992–1.004	0.55	n.a		

Bolded text signifies significant findings

Using multivariate regression model analysis, we showed that the long-term drainage period remained a significant independent risk factor for major postoperative complications (CDC > 2) as well as periampullary cancer in our cohort. The odds ratio for PBD II was 3.386 (95% CI: 1.507–7.606; *p* < 0.01) and 5.519 for periampullary carcinoma (95% CI: 1.722–17.685; *p* < 0.01) (Table 5).

### Secondary endpoint

**Fig. 1:**
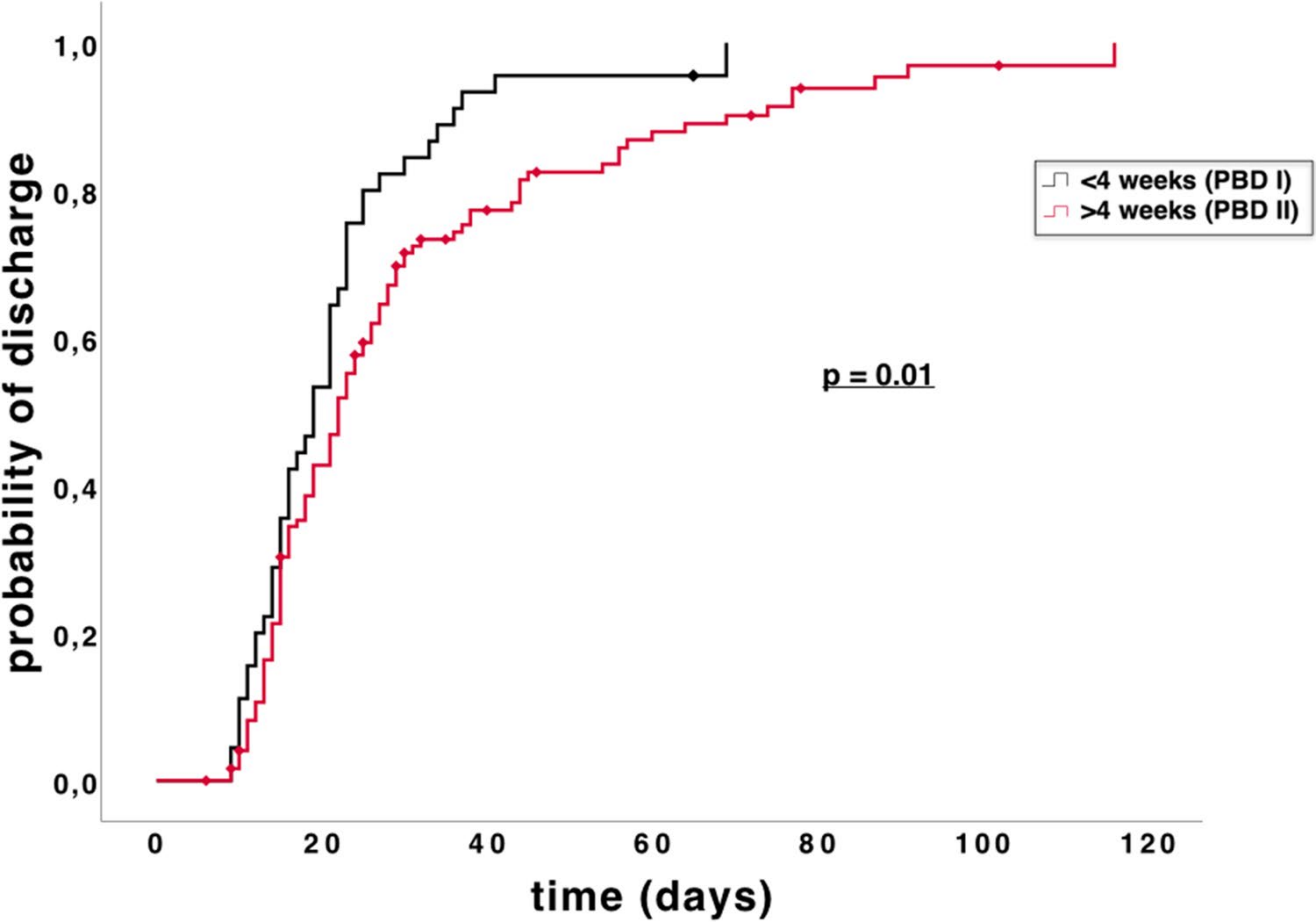
Kaplan–Meier analysis of time to discharge for all patients: Patients in the PBD I group were discharged significantly earlier than those in the PBD II group (*p* = 0.01)

The median LOS was 21 days (IQR 14–29.3), and a significant difference is observable between the groups (*p* = 0.02). The 30-day rehospitalization rate was 13.5%. By performing a log-rank test for the probability of discharge following PD, we found that patients in the PBD I group were significantly more likely to be discharged from the hospital earlier (*p* < 0.01) (Fig. 1).

**Fig. 2:**
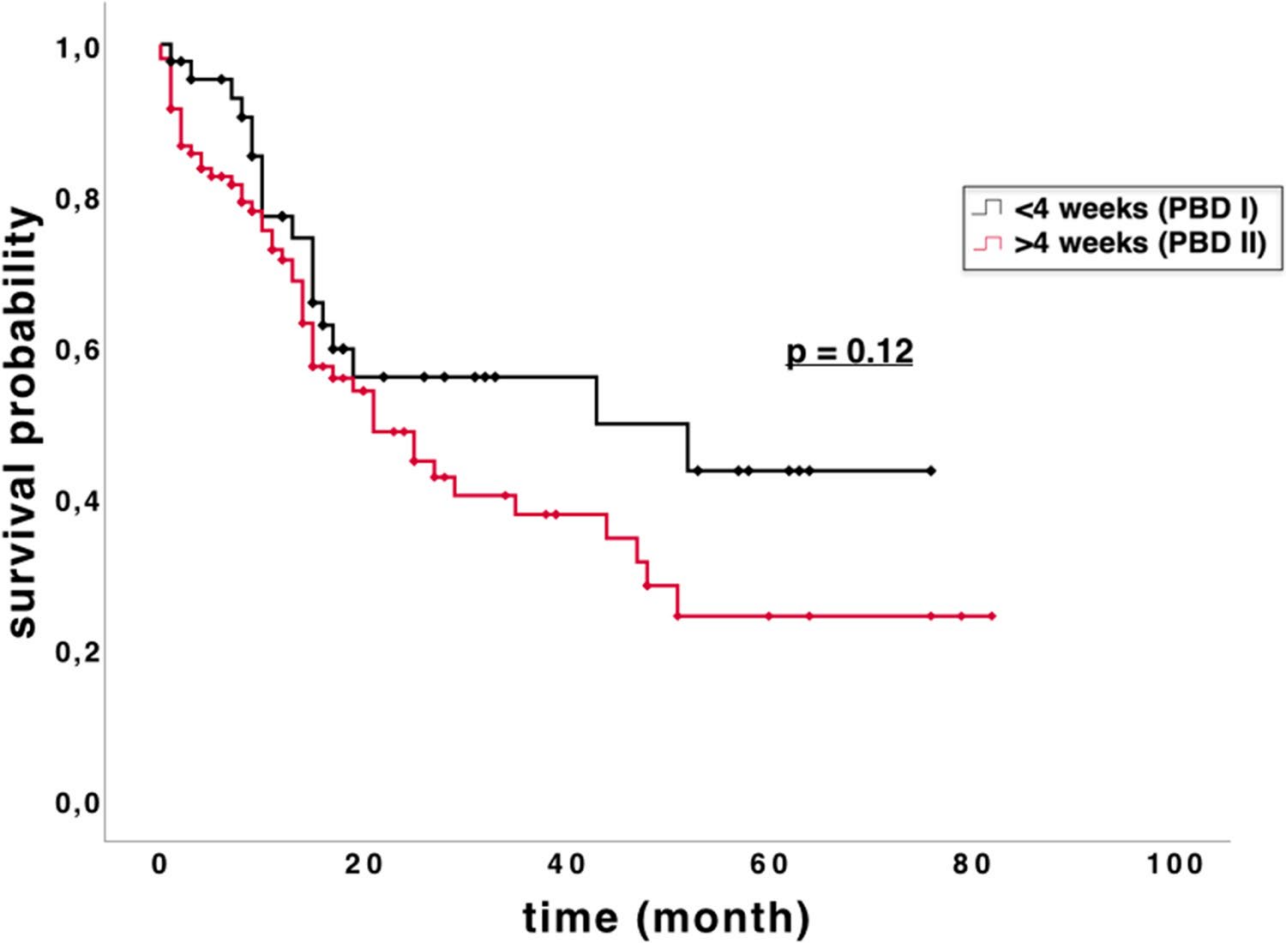
Kaplan–Meier analysis of the overall survival for all patients: No statistically significant difference in overall survival was found between PBD I and PBD II

OS adjusted for underlying disease entity for the PBD I and PBD II groups was 44.1 months and 34.6 months, respectively (Table 5, Fig. 2).

## Discussion

This retrospective study provides data on the potential correlation of different PBD durations to postoperative morbidity, mortality, LOS, and survival rate following pancreatic surgery. In this study, we included 170 patients who underwent PBD. This cohort represented 24.2% of all patients who were scheduled for pancreatic surgery between January 2012 and June 2018 at our high-volume center. Previous trials reported preoperative drainage rates of 30–63% for high-volume pancreatic centers [4, 11, 19–21]. Reducing the frequency of PBD leads to fewer complications [1, 21, 22] and may also reduce time to surgery. Whether this is favorable for patients in need of pancreatic resection remains controversial [7, 23].

Current guidelines for managing cholestasis in patients in need of pancreatic resection (either for an underlying malignancy or chronic pancreatitis) recommend that patients should only be considered for PBD if the preoperative bilirubin level exceeds 250 µmol/L, cholangitis is present, or if the patient is undergoing preoperative chemotherapy [5]. The accurate and consistent application of this recommendation would likely result in fewer patients undergoing PBD. However, the results of international trials indicate that 40% of patients scheduled for pancreatic surgery receive PBD unnecessarily [3, 20, 24]. Furthermore, approximately 60–75% of patients receive PBD before surgical consultation [3, 24]. Therefore, it is reasonable to expect that most patients will continue to undergo PBD prior to PD. Thus, further investigations are needed to determine the appropriate duration of PBD in this highly vulnerable patient cohort.

In our cohort, 80 patients (47.1%) experienced a major complication (CDC > 2) during the postoperative follow-up. These data correspond to the complication rates reported in previous studies that have investigated the influence of PBD on postoperative morbidity [20, 25]. However, if the time interval of PBD is considered, the complication rate between groups is much more differentiated. Using this approach, our univariate and multivariate analyses indicated that patients in PBD I experienced significantly fewer complications compared to those in PBD II. In line with these findings, the postoperative mortality was substantially higher among patients in PBD II, although this result was not significant.

In addition, the median hospital stay duration of the short-term PBD group (19 days) was significantly shorter than that of the long-term PBD group (22 days). Using the log-rank test, we found a significantly greater probability of postoperative early hospital discharge for patients in the short-term PBD group. Moreover, after adjusting for the indication for adjuvant chemotherapy, more patients in the short-term group (51.2%) received adjuvant chemotherapy than those in the long-term group (35.3%).

The complexity of determining the appropriate PBD time is evident from these contradictory data. While our data suggest that a shorter PBD time might be beneficial in terms of postoperative morbidity, Sandini et al. concluded that pancreatic resection should be postponed for 4–6 weeks after stent placement to reduce postoperative complications. These conclusions are supported by experimental data showing that impaired liver cell function due to cholestasis is restored 4–6 weeks after biliary drainage at the earliest [26–28]. In contrast, another trial published by Son et al. [10] concluded that the interval between PBD and pancreatic resection should be as short as 2 weeks. A possible explanation for the proposed shorter PBD time might be the change in bacterial flora in the bile that occurs with a longer duration of PBD and the complications (e.g., occlusion, cholangitis, or dislocation) associated with a biliary stent. Our data support the latter assumption, as we found that the short-term stenting group had significantly fewer positive bile duct cultures compared to the long-term stenting group.

The strength of the presented dataset is that it originates from a single high-volume center with uniform standards concerning the decision for PBD [5], along with the route and type of drainage.

Notably, while our study provides some new evidence, we acknowledge its limitations. In line with the results reported by Son et al. and Sandini et al. [11], we present a heterogeneous group of patients and indications for pancreatic resection, which may affect the transferability of the presented results. Even though standards concerning the decision for PBD exists at our institution, we acknowledge that some patients which were transferred from other hospitals might have received the stent with a different standard for placement (e.g., type of stent). Especially the type of stent is a significant variable in terms of preoperative stent-associated complications. Recent evidence suggests that PBD should be carried out with metal stent as this shows a reduction of stent-related complications while the postoperative complications remain unaffected.

Furthermore, the retrospective design of the study has inherent limitations, such as recall and selection biases. Moreover, the severity of the underlying disease might also influence the PBD-time which is not possible to assess in this retrospective design.

Another limitation we need to acknowledge is the distribution of the different types of histo-pathology between the groups: Even though the allocation of malignant and benign disease was comparable between the groups, still the different pathologies are not distributed completely equal between the PBD groups. This still can cause a bias in the survival analysis.

Nevertheless, discussion on the appropriate duration of preoperative drainage is still ongoing. Given the inconsistent evidence, the question of whether postponing PD in cases of PBD increases postoperative morbidity has not yet been answered. However, one must be mindful of the psychological burden of these patients and pancreatic resection should be performed at the earliest possible stage, unless stronger contrary evidence arises.

## Conclusion

PBD in patients scheduled for pancreatic surgery is associated with substantial postoperative morbidity and a high mortality rate. In contrast to recently published results by Sandini et al., [11] we showed that a short PBD time is superior in terms of postoperative morbidity with a comparable OS rate. Unless stronger evidence becomes available, pancreatic resection should be performed at the earliest possible stage after PBD.

Admittedly, the complexity of determining the appropriate preoperative drainage time in light of the increasing number of patients being treated in a neoadjuvant setting cannot be determined with small, inconsistent retrospective trials. In view of these contradictory results, we advocate performing a prospective multicenter trial to determine the optimal interval between PBD and time-to-surgery.

## Data Availability

On request.
